# Cu_3_As: Uncommon Crystallographic Features, Low-Temperature Phase Transitions, Thermodynamic and Physical Properties

**DOI:** 10.3390/ma16062501

**Published:** 2023-03-21

**Authors:** Marianne Mödlinger, Alessia Provino, Pavlo Solokha, Federico Caglieris, Michele Ceccardi, Daniele Macciò, Marcella Pani, Cristina Bernini, Dario Cavallo, Andrea Ciccioli, Pietro Manfrinetti

**Affiliations:** 1 Department of Chemistry, University of Genoa, 16146 Genoa, Italy; 2 Department of Physics, University of Genoa, 16146 Genoa, Italy; 3 Institute SPIN-CNR, 16152 Genoa, Italy; 4 Department of Chemistry, Sapienza University of Rome, 00185 Rome, Italy

**Keywords:** copper arsenides, lonsdaleite sublattice, X-ray diffraction, first-order structural transition, differential scanning calorimetry, electrical resistivity, magnetic susceptibility

## Abstract

The formation and crystal structure of the binary Cu_3_As phase have been re-investigated. Some physical properties were then measured on both single crystal and polycrystalline bulk. Cu_3_As melts congruently at 835 °C. At room temperature (RT), this compound has been found to crystallize in the hexagonal Cu_3_P prototype (*hP*24, *P*63*cm*) with lattice parameters: *a* = 7.1393(1) Å and *c* = 7.3113(1) Å, rather than in the anti HoH_3_-type (*hP*24, *P*–3*c*1) as indicated in literature. A small compositional range of 74.0–75.5 at.% Cu (26.0–24.5 at.% As) was found for samples synthesized at 300 and 400 °C; a corresponding slight understoichiometry is found in one out of the four Cu atomic sites, leading to the final refined composition Cu_2.882(1)_As. The present results disprove a change in the crystal structure above RT actually reported in the phase diagram (from γ’ to γ on heating). Instead, below RT, at T = 243 K (−30 °C), a first-order structural transition to a trigonal low-temperature superstructure, LT-Cu_3−x_As (*hP*72, *P*–3*c*1) has been found. The LT polymorph is metrically related to the RT one, having the *c* lattice parameter three times larger: *a* = 7.110(2) Å and *c* = 21.879(4) Å. Both the high- and low-temperature polymorphs are characterized by the presence of a tridimensional (3D) uncommon and rigid Cu sublattice of the lonsdaleite type (Cu atoms tetrahedrally bonded), which remains almost unaffected by the structural change(s), and characteristic layers of triangular ‘Cu_3_As’-units (each hosting one As atom at the center, interconnected each other by sharing the three vertices). The first-order transition is then followed by an additional structural change when lowering the temperature, which induces doubling of also the lattice parameter a. Differential scanning calorimetry nicely detects the first low-temperature structural change occurring at T = 243 K, with an associated enthalpy difference, ΔH_(TR)_, of approximately 2 J/g (0.53 kJ/mol). Low-temperature electrical resistivity shows a typical metallic behavior; clear anomalies are detected in correspondence to the solid-state transformations. The Seebeck coefficient, measured as a function of temperature, highlights a conduction of *n*-type. The temperature dependence of the magnetic susceptibility displays an overall constant diamagnetic response.

## 1. Introduction

The Cu-As alloys, which constitute the commonly known “arsenical bronzes”, were the first alloys intentionally produced by mankind. Arsenical bronzes were used in the fabrication of weapons, tools and jewelry during the Copper and Bronze Age. The Cu-As alloys appeared in the archaeological record as early as ca. 5000 BCE on the Iranian plateau and later in Central Europe in the fourth and early third millennium BCE [1]. Over time, the Cu-As alloys were progressively replaced by Cu-Sn (tin bronze), Cu-Pb-Sn (lead-tin bronze), and finally Cu-Zn (brass) alloys. The Cu-As alloys were no longer used until the 19th century. Their use started again only with the development of the steam engines, as the high corrosion resistance of the Cu-As alloys compared to that of other available materials made them the ideal alloys for applications. Once steam vessels became obsolete, Cu-As alloys were basically no longer used [2]. While the investigation of Cu-As alloys is essential to archaeologists to better understand the beginnings of metallurgy, the study of their crystal chemistry, thermodynamic stability and physical properties is fundamental for both potential technological applications and further understanding the metallurgy behind their usages in the past. In this regard, although the Cu-As binary phase diagram has been the subject of numerous investigations during the last century, it still lacks detailed studies, and most of it is reported with uncertainty. Only a few Cu-As compounds are known to form; however, for most of these binary compounds, neither the crystal structure (and so the true stoichiometry), nor the existence range in the phase diagram or the physical properties have been determined.

The most recent work on the Cu-As phase diagram is an assessment collecting the results of several studies carried out throughout the last century [3]. The Cu-As binary phases accepted from current literature data are as follows: Cu_3_As, which forms congruently from the liquid (L) at 827 °C and at the composition of 26.25 at.% As (Cu_3_As ⇆ L); the metastable Cu_5_As_2_ phase, which exists in the temperature range from about 300 to 709 °C, forming by peritectic reaction between Cu_3_As and liquid at ≈709 °C (Cu_3_As + L ⇆ Cu_5_As_2_) and decomposing eutectoidally into Cu_3_As and As at 300 °C (Cu_5_As_2_ ⇆ Cu_3_As + As); and the Cu_2_As phase, which is reported as a metastable phase with no details about its formation [3,4]. In the above assessment, both Cu_3_As and Cu_5_As_2_ are reported to exist within a range of solid solubility (2–3 at.% As the former and 1–2 at.% As the latter) and with two structural modifications: γ and γ′ for the former, and δ and δ′ for the latter compound. The γ and γ′ phases are the high-temperature (HT) and low-temperature (LT) forms for Cu_3_As, respectively, while δ and δ′ are high-temperature (HT) and low-temperature (LT) forms for Cu_5_As_2_, respectively. The γ phase (HT form of Cu_3_As) was reported to be isotypic with the hexagonal Na_3_As-type (Pearson’s symbol *hP*8, space group *P*6_3_*/mmc*, No. 194) [5]. Its lattice parameters are *a* = 4.17 Å and *c* = 7.32 Å at 490 °C [3,6]; this is the only report on the existence of a HT form for the Cu_3_As compound. The γ phase undergoes an allotropic transformation to the γ′ phase between 450 and 475 °C [3]. On the other hand, the LT modification of Cu_3_As, γ′, is reported to adopt the trigonal Cu_3_As-type (or HoH_3_ or anti-LaF_3_ [7]; *hP*24, *P–*3*c*1, No. 165) with lattice parameters *a* = 7.124 Å and *c* = 7.296 Å [3,6]. In a subsequent work by X-ray single crystal diffraction technique, Cu_3_As as mineral domeykite was also reported to crystallize in a cubic Cu_3_As-type structure, derivative of the W-type (*cI*64, *I−*43*d*, No. 220) [8]. The structure of Cu_3_As in all other studies was determined only by X-ray powder diffraction of samples annealed at relatively high temperature. However, none of the two crystal structures for Cu_3_As have ever been confirmed. As for the Cu_5_As_2_ compound, it is only reported with an uncertain crystal structure and stoichiometry. A possible prototype for the LT form of Cu_5_As_2_, δ′, was proposed to be the orthorhombic Mg_5_Ga_2_-type (*oI*28, *Ibam*, No. 72, [9]). However, to date, the true crystal structure of this phase (and thus its exact stoichiometry) has never been determined to date, nor has the existence of its HT form been confirmed.

The Cu_3_As compound remains the ‘most well-known’ phase in the Cu-As binary system; it is used in semiconductors and photo-optic applications [10]. Nevertheless, although it has been reported to crystallize in two allotropic forms depending on temperature, none of them has ever been established with certainty. In our work, we have investigated the Cu_3_As intermetallic in detail. Specifically, we have investigated its formation and thermodynamic stability, its crystal structure both at high and low temperature, and some of its physical properties.

## 2. Experimental Methods

### 2.1. Synthesis

Samples with a nominal composition Cu_3_As (for a total mass of 6–8 g each) were prepared by vapor-solid reaction between Cu and As. The starting metals used were Cu in small pieces (99.997 wt.% purity, Metallwerke Brixlegg, Brixlegg, Austria, MB-OF101 certified) and As in lumps (99.99 wt.% purity, Alfa Aesar, Haverhill, MA, USA). In order to remove traces of oxides, before use, Cu pieces were treated in nitric acid (5–10%), then rinsed with water and finally with absolute ethanol. For the same purpose, As lumps were sealed under vacuum in a Pyrex tube and heated up to 300 °C while keeping the top end of the tube right outside the furnace entrance. This treatment allowed As_2_O_3_ vapors to migrate towards the far cold end of the tube and finally condense. The so pre-treated elements, weighed in adequate stoichiometric amounts, were sealed inside a quartz tube under vacuum and slowly heated (in steps of 40 °C above 250 °C) in an electric furnace until the desired temperature was reached. The samples were prepared at several temperatures between 300 °C and 750 °C; at the end of the heat treatment, most of the samples were slowly cooled within the furnace, some were quenched in air, and a few of them were quenched in water. The alloys were left inside the quartz tube until analyses were carried out to prevent possible oxidation. The final samples were gray in color and featured by large crystals. An overview on binary alloys prepared, their thermal treatments and analyses carried out is provided in Table S1.

### 2.2. Sample Characterization

The microstructure of the alloys was checked using both light optical microscopy (LOM), utilizing a Leica DM1750M (Wetzlar, Germany) in both bright field and polarized light (with up to a 1000× magnification), and scanning electron microscopy (SEM) complemented with a microprobe for semiquantitative analysis (EDX), utilizing a Leica Cambridge 360 microscope equipped with an Oxford X-Max 20 analyzer (with work parameters set to EHT 20.0 kV and probe current 220 pA; Oxford Aztec software, Abingdon, UK). The metallographic specimens were prepared using a standard polishing technique, embedded in cold-mounting acrylic resin, grinded with abrasive papers (up to 1200 mesh), and finally polished with diamond paste down to 1 μm. Pure Co metal was used for calibration, and the overall area of the specimen (15–20 mm^2^) was used as a standard of known composition to improve the accuracy of the measurements, estimated to be of ±1.0 at.% for each element. The compositional analysis was performed using characteristic X-ray intensities of each element, with an acceleration voltage of 20 KV and an acquisition time of 100 s.

### 2.3. Structural Characterization

Structural characterization was performed using both powder and single-crystal X-ray diffraction (XRD) techniques. Powder X-ray patterns (PXRD) were collected on a Bruker D4 Endeavour diffractometer (Cu Kα radiation) equipped with an area detector (generally within 2ϑ ranges of 5–100°, with a step size of 0.02° and a counting time of 4 or 6 s/step). Pure Si was used as an internal standard (*a* = 5.4308(1) Å). Powders were prepared by grinding polycrystalline pieces and placing them on a single-crystal Si zero background sample holder. The patterns were indexed using Lazy Pulverix [11] software, and accurate lattice parameters were calculated using least-square methods (handmade software). Rietveld structural refinements were carried out on most of the samples using FullProf software [12].

Good-quality single crystals were selected from crushed specimens, affixed to a glass fiber with grease, and checked by X-ray diffraction. Temperature-dependent screening measurements were obtained routinely on a three-circle Bruker D8 QUEST diffractometer equipped with an Oxford Cryostream 1000. The PHOTON III 14 photon counting detector equipped with graphite monochromatized Mo Kα (λ = 0.71076 Å) radiation is mounted on the diffractometer. Data collection strategies (composed of ω- and φ-scans) were calculated by APEX4 engines [13]. Intensity data were collected over the reciprocal space up to ≈45° in θ (achieving a resolution of ≈0.5 Å) with exposures of 15–20 s per frame. Successively, data were reduced using SAINT [14] and XPREP [15]. Lorentz, polarization and absorption effects were corrected using SADABS [16]. The crystal structures were solved and refined with the aid of SHELXL-2019/1 [17].

### 2.4. Thermal Analysis

Samples with a mass of 0.65–0.75 g were cut and used for differential thermal analysis (DTA) (NETZSCH DTA 404 S). The samples were placed in Al_2_O_3_ crucibles and covered with an Al_2_O_3_ lid, and an additional cap of pure Cu foil (6N) aimed to capture small amounts of As-vapor that could be released. Each sample was heated under pure Ar at about 100 °C above its melting temperature (previous observation of the sample during synthesis already gave a good indication of the approximate melting temperature +/− 50 °C) and usually held at the selected temperature for 5 min. Heating and cooling rates were usually chosen at 10 and 5 K/min, respectively. For the measured temperatures, the accuracy was estimated to be within ±5 °C. No contamination of the alloy by the crucible material was observed by SEM-EDX.

Below room temperature differential scanning calorimetry (DSC) was employed, using a TA Instruments DSC250 Discovery under an N_2_ flux of 50 mL/min. Heating and cooling runs were performed at 5 or 10 °C/min, spanning a temperature range from −80 to 40 °C. The analyses were carried out on both single-crystalline and polycrystalline bulk samples of about 10–30 mg in Al crucibles.

### 2.5. Transport and Physical Property Measurements

Electrical resistivity and Seebeck effect were measured on properly shaped Cu_3_As single crystals using the commercial apparatus Physical Properties Measurement System (PPMS, Quantum Design) with homemade sample holders. The electrical resistivity was measured in a standard four-probe configuration, experimentally realized with copper leads glued to the sample through silver paint, in a temperature range between 2 and 310 K and magnetic field up to 9 Tesla. In the Seebeck effect setup, one side of the sample was anchored to a thermal mass, while a resistive heater (R = 2.8 kΩ) was glued to the other side in order to generate a temperature gradient. A calibrated Chromel-Au-Chromel thermocouple was used to measure the temperature gradient across the sample, while two copper electrodes were attached to the sample to pick up the Seebeck voltage. The Seebeck data have been collected between 290 and 15 K.

Magnetic susceptibility was measured both on a single crystal and on a polycrystalline sample of Cu_3_As using the commercial apparatus SQUID (MPMS, Quantum Design). The temperature-dependent magnetization measurements were acquired in external magnetic fields of 1 T, from 5 to 300 K.

## 3. Results and Discussion

### 3.1. Phase Analysis

**Figure 1:**
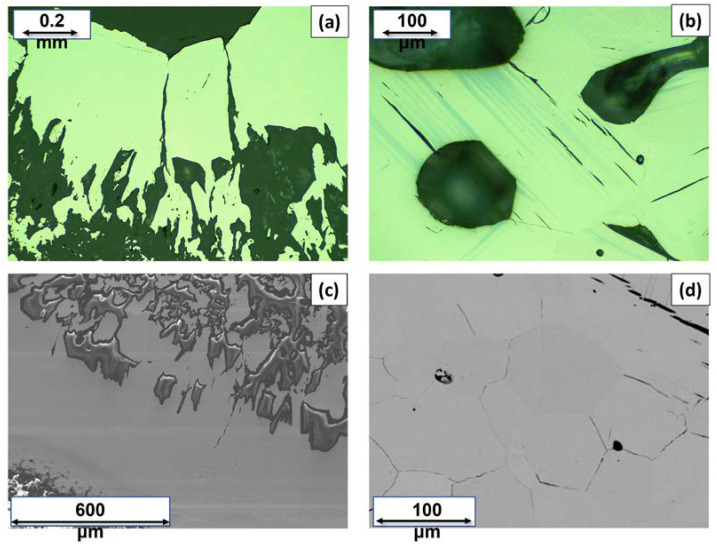
LOM images of the Cu_3_As samples synthesized at 350 °C—13 days (**a**) and at 500 °C—16 days (**b**). SEM microphotographs in Back-Scattered-Electron (BSE) mode of the same samples: 350 °C—13 days (**c**) and 500 °C—16 days (**d**).

**Figure 2:**
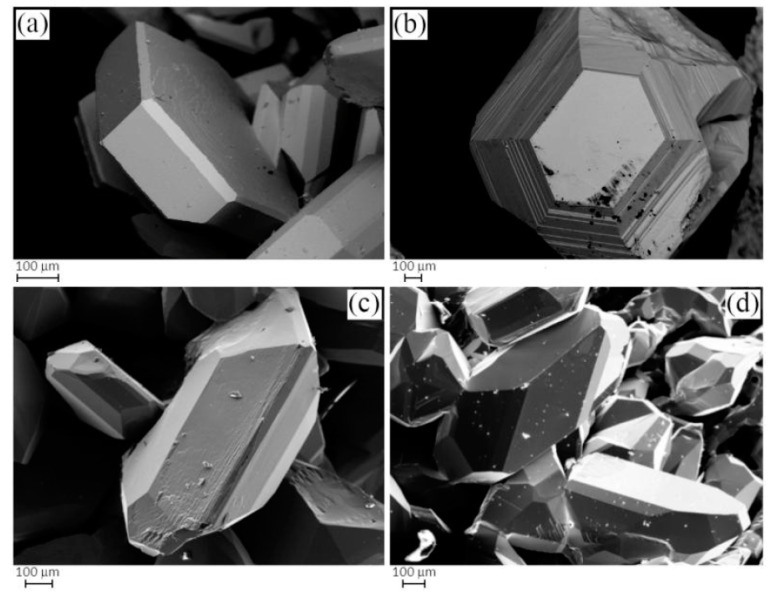
SEM microphotographs in Secondary Electron (SE) mode of selected samples; (**a**,**b**) show crystals grown after thermal treatment at 300 °C—20 days, while (**c**,**d**) show crystals grown after thermal treatment at 400 °C—14 days.

For simplicity, from now on, in order to describe and identify each Cu_3_As sample in the discussion, we will refer to their thermal treatment. The Cu_3_As samples prepared at 350 °C/13 days, 400 °C/5 days, 400 °C/14 days and 650 °C/19 days/Quenched were practically single-phase containing the Cu_3_As compound. This was confirmed by both SEM-EDX and powder X-ray diffraction; see Table S1 for more details on their synthesis conditions and phase analysis. Figure 1a,b show the light optical microscope (LOM) images of the samples heat-treated at 350 °C/13 days and 500 °C/16 days, respectively. While the former appears to be single-phase (Figure 1a), the latter contains small amounts of an extra phase present as stripe-like darker grains (Figure 1b). The corresponding backscattered images, as obtained by SEM technique, are shown in Figure 1c,d, respectively. Notable is the difference in their microstructure: while the former sample still shows single crystals of the same phase with different size growing next to each other, the latter already shows a polycrystalline bulk structure, with several pairs of parallel strips of ‘Cu_5_As_2_’ (composition as confirmed by EDX). Figure 2a,b show SEM microphotographs of large crystals grown in the Cu_3_As sample 300 °C—20 days, while Figure 2c,d show some crystallites from the Cu_3_As sample after annealing at 400 °C/14 days. Interesting and very peculiar are the large hexagonal-shaped crystals, which are typical of the symmetry of this compound. EDX analysis was carried out on both areas and points for the samples: 300 °C/20 days, 350 °C/13 days, 500 °C/16 days, 750 °C/15 days. The data, overall, suggest a compositional range of 74.0–75.5 at.% Cu (26.0–24.5 at.% As) for Cu_3_As.

### 3.2. Single-Crystal Structural Study of the Cu_3_As Compound

The single-crystal investigation was carried out on high-quality crystals picked up from the sample 350 °C/3 days. Diffraction images were recorded at low temperature (LT) from −38 °C (235 K) to −118 °C (155 K) at steps of 5 °C, while complete datasets were recorded at ambient (room temperature, RT) conditions and at T = −78 °C (195 K). The temperature-dependent diffraction data indicated the presence of two low-temperature phases.

#### 3.2.1. RT-Cu_3_As Crystal Structure

The results from present work prove the true crystal structure of Cu_3_As to be of the hexagonal Cu_3_P-type (*hP*24, *P*6_3_*cm*, No. 185), and not of the hexagonal anti HoH_3_-type. Moreover, we also find that Cu_3_As does not exist with other high-temperature modifications, at least up to 750 °C. The crystal structure of the Cu_3_P prototype was investigated by the single-crystal technique by Olofsson [18]. This structure shows five symmetry inequivalent Wyckoff sites; four of these positions (two with 6*c* symmetry, one with 4*b* symmetry, and one with 2*a* symmetry) are occupied by Cu atoms, and one site (with 6*c* symmetry) is occupied by P atoms. A vacancy was observed on all the Cu sites, which resulted in the final refined stoichiometry Cu_2.82_P [18]. A more recent work [19] also reported this phase to be under-stoichiometric. It must be noted that while in the former work, the Cu:P ratio was determined by X-ray diffraction technique (both on single crystal and powder) [18], in the latter work, the Cu:P ratio was determined only by X-ray photoelectron spectroscopy (XPS) technique, without any X-ray diffraction investigation [19]. Moreover, Reference [18] also reveals a range of solid solubility going between Cu_2.82_P and Cu_2.73_P; never reaching the full 3:1 stoichiometry. All the other literature data on Cu_3_P report this phase to be fully stoichiometric [7]. Therefore, for simplicity, from now on, we will refer to this structural prototype as Cu_3_P. Several compounds are reported to adopt the Cu_3_P-type: Na_3_As, Cs_3_As, Na_3_Au, Mg_3_Au, Cd_3_Au, K_3_Bi, LaF_3_, (La_1−x_Pr_x_)F_3_, UF_3_, U_0.75_F_3_, Mg_3_Ir and Mg_2.8_Ir, IrIn_3_, Mg_3_Pd, Mg_3_Rh, Na_3_N, YH_3_ [7].

A total of 2570 frames were collected. The integration of the data using a hexagonal unit cell yielded a total of 18,668 reflections, of which 988 were independent (average redundancy 18.8, completeness = 100.0%, R_int_ = 7.48%, R_sig_ = 2.55%). Analysis of the systematic absences conditions for the RT dataset is consistent with three hexagonal space groups (*P*6_3_/*mcm*, *P*–6*c*2 and *P*6_3_*cm*) and two trigonal ones (*P*–3*c*1 and *P*3*c*1). In all these space groups, the structure solution resulted in roughly the same structure model; however, refinements in centrosymmetric space groups show noticeably higher residuals and thermal ellipsoids for Cu atoms strongly elongated/flattened. The structural models in *P*6_3_*cm* and *P*3*c*1 are group-subgroup related and have no significant atoms shifts, thus, the non-centrosymmetric *P*6_3_*cm* structural model is considered as the correct one. The RT-Cu_3_As compound is isostructural with the Cu_3_P prototype; the unit cell contains 24 atoms, with As atoms located in a 6*c* site, and Cu atoms distributed among two 6*c*, 4*b* and 2*a* positions. The refinement of the structural model went smoothly, showing no Cu/As statistical mixture; instead, the site occupation factor of Cu4 site (6*c*, *x* = 0.368 *y* = 0 *z* = 0.413), refining to ca. 0.9, induced further reduction in residuals. After considering the presence of the inversion twin, the final anisotropic refinement converged to comparably good residuals complemented by a flat difference Fourier map (see Table 1). The final standardized atomic coordinates and equivalent displacement parameters (*U*_eq_) are collected in Table 2, while anisotropic displacement parameters are in Table 3. The corresponding CIF file, available in the Supporting Information, has been deposited at the Cambridge Database with the following depository number: CSD-2235455.4

Interestingly, it can be observed that the room temperature structure of the Cu_3_As compound (RT-Cu_3−x_As) is formed by two distinct parts: (1) a 3D network built up by Cu2 and Cu3 atoms only, (2) slightly undulated 2D layers of interconnected triangular ‘Cu_3_As’ units, each formed by only Cu1 and Cu4 atoms at the three vertices and hosting one As atom at the center.

**Figure 3:**
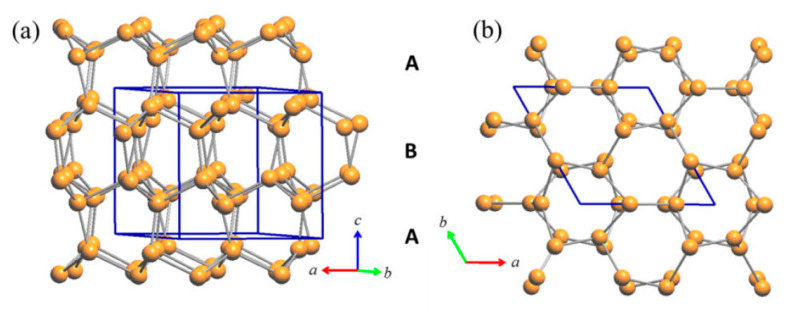
(**a**) Perspective view of the rigid Cu sublattice of the lonsdaleite type formed in the RT-Cu_3_As phase by the contribution of the Cu2 and Cu3 atoms only; (**b**) top view (along the *c*-axis) of the same lonsdaleite sublattice.

The tridimensional network of Cu2 (site 6*c*) and Cu3 (site 4*b*) atoms represents an uncommon and peculiar sublattice of Cu atoms of the lonsdaleite type. Lonsdaleite, also known as *hexagonal diamond* (also called *meteor* or *impact diamond*), is generally accepted by the scientific community as one of the carbon polymorphs, in addition to *hexagonal graphite* and *cubic diamond*. However, despite the conditions of its appearance and its structural peculiarities, it remains a bit controversial and questioned, as it is considered as *cubic diamond* dominated by extensive stacking faults and twins [20]. Indeed, at the present time, pure lonsdaleite has never been found or synthesized as single crystal or in bulk form; and its presence, either from natural origin or produced artificially, namely synthesized in a laboratory under thermobaric (high dynamic/static pressure—high temperature, HP-HT) conditions [21,22,23,24,25,26,27,28,29,30], has been usually observed only in small amounts and as nanoparticles inside diamond crystals. Similarly to the structure of both cubic diamond and hexagonal lonsdaleite, in which each *sp*3-hybridized carbon atom is bonded to four other carbon atoms, Cu2 and Cu3 atoms form a tetrahedral structure resulting in a 3D rigid network with strong chemical bonds. The four Cu2–Cu3 bond distances are 2.531 Å, 2.672 Å, 2.672 Å and 2.728 Å, which are well comparable to the bond lengths in elemental Cu [31] (see Figure 3). Moreover, Cu atoms filling these two sites (6*c* and 4*b*) form puckered layers with six-membered rings arranged in an armchair-like configuration (Figure 3a).

**Figure 4:**
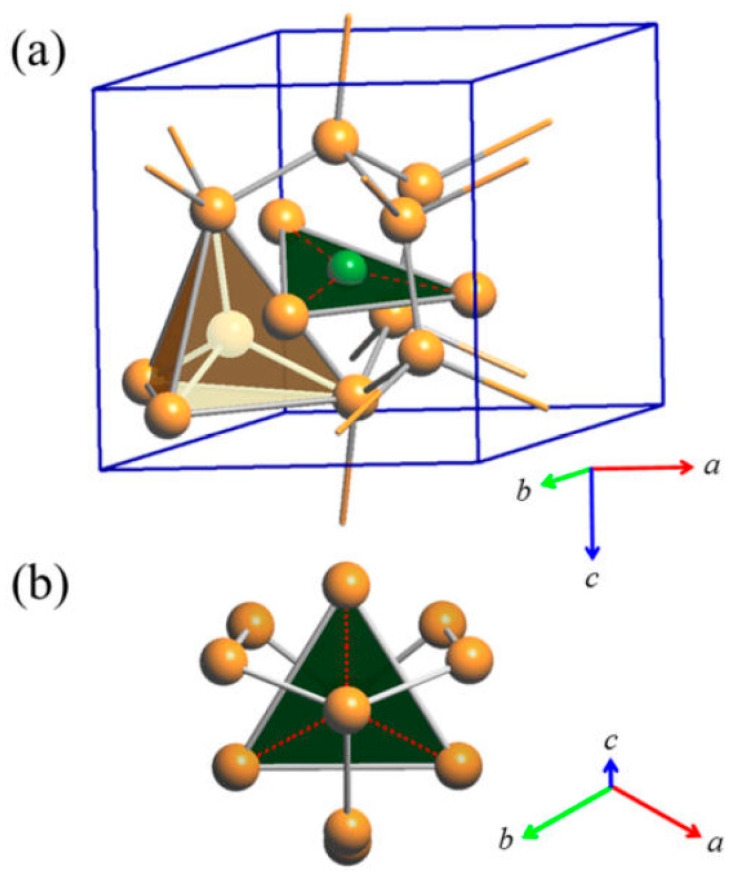
(**a**) Triangular ‘Cu_3_As’ unit (Cu4–Cu1–Cu4) hosting one As atom (6*c*) and placed inside one three-rings cage of the first tridimensional Cu sublattice. One of the tetrahedral units [Cu@Cu_4_] formed by all the Cu atoms pertaining to the lonsdaleite sublattice (Cu2 and Cu3 atoms) is also shown; (**b**) top view of one triangular ‘Cu_3_As’ unit. Orange and green balls indicate Cu and As atoms, respectively.

**Figure 5:**
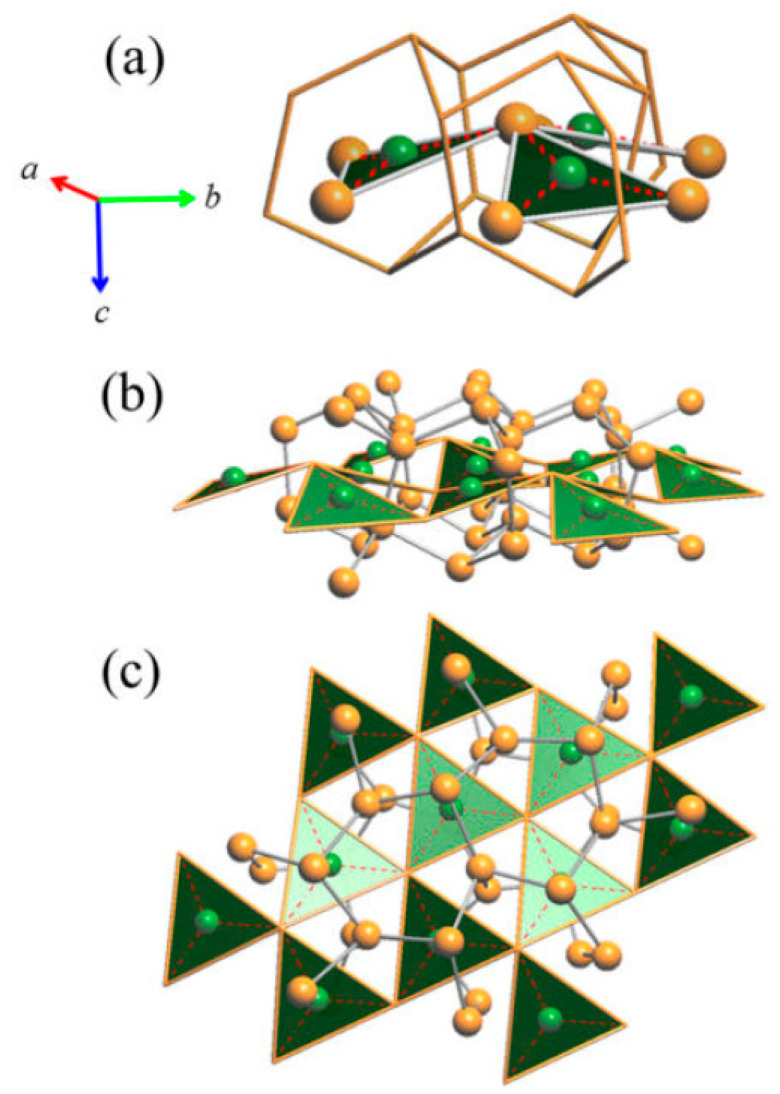
(**a**) Three triangular units sharing one vertex. (**b**) Perspective view of a layer formed by the ‘Cu_3_As’-triangular units. (**c**) Top view of the layer shown in (**b**). Note: for simplicity, in (**b**) and (**c**) the Cu atoms at the vertices of the ‘Cu_3_As’ units (Cu4–Cu1–Cu4) are not shown. Orange and green balls indicate Cu and As atoms, respectively.

**Figure 6:**
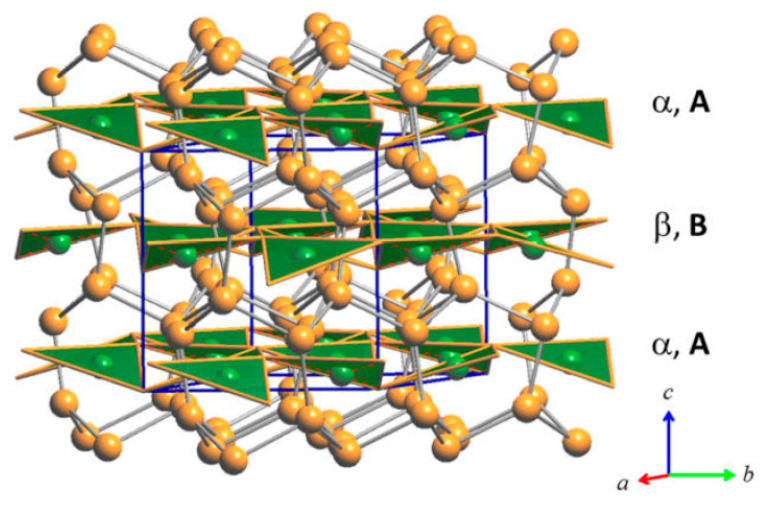
Perspective drawing of the RT structure of the Cu_3_As compound. Each unit cell contains two layers, α and β, of vertex-sharing triangular ‘Cu_3_As’ units. Orange and green balls indicate Cu and As atoms, respectively.

**Figure 7:**
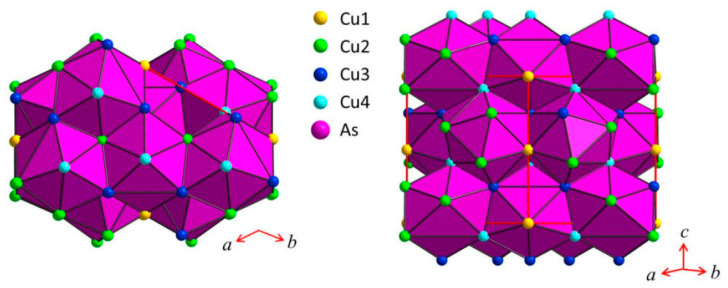
Sketch of the crystal structure of Cu_3_As where the coordination polyhedra around As atoms, As@Cu_11_, are highlighted.

The triangular ‘Cu_3_As’ units are formed by one Cu1 (2*a*) and two Cu4 (6*c*) atoms placed at the three vertices of an isosceles triangle (with angle values of about 57.6° and 61.2° for the <Cu4–Cu1−Cu4> and <Cu4−Cu4−Cu1> angles, respectively). Each triangle hosts one As atom (in site 6*c*) centered onto the plane (with bond lengths of 2.460 Å and 2.436 Å, for the As–Cu1 and the two As–Cu4 bonds, respectively) and sets inside one three-rings cage of the previously described tridimensional Cu sublattice (Figure 4). Such triangles are interconnected three by three by sharing one Cu atom at the vertex, arranged slightly tilted each other but all facing to the same versus (Figure 5a), to finally form a structural layer (Figure 5b,c). The layers, in a number of two per unit cell and labelled α and β, are placed perpendicular to the hexagonal *c* axis, with the Cu atom interconnecting three ‘Cu_3_As’ units centering the columnar cavities available along the *c* axis of the Cu sublattice (lonsdaleite-type network). The interlayer distance between layers α−β is 3.653 Å. The interatomic distances corresponding to the first coordination sphere (distances for which d_obs_/Σ*r*_M_ ≤ 1.16, where d_obs_ is the interatomic distance and Σ*r*_M_ is the sum of the two metallic radii) are in Table 4; the structure does not present direct interactions As−As. Overall, the resulting crystal structure is shown in Figure 6. On the other hand, the structure of the RT-Cu_3_As phase could be also described as a complex array of pseudo Frank-Kasper polyhedra, with composition As@Cu_11_, connected with each other by sharing their triangular faces. Figure 7 shows a sketch of the structure where these polyhedra around As atoms are highlighted.

**Figure 8:**
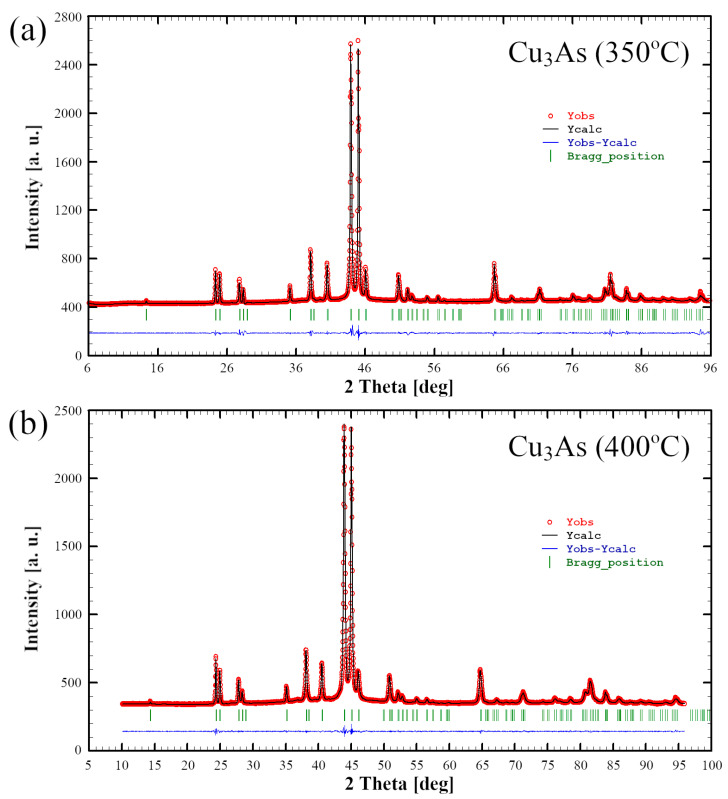
Rietveld refinement profiles (black line) obtained for the Cu_3_As samples 350 °C—13 days (**a**) and 400 °C—14 days (**b**). The observed powder patterns are highlighted in red. The lower profile (blue line) gives the difference between observed and calculated data; the Bragg angle positions are indicated by vertical bars (green).

Results from Rietveld refinement carried out on powders from the samples 350 °C/13 days and 400 °C/14 days further corroborate well with the data obtained from single-crystal analysis (Figure 8). Refinement data are collected in Tables S2 and S3. No structural changes between 400 and 650 °C are observed. This result disproves the literature data, which give a structure transition from γ’–γ within this temperature range [3].

#### 3.2.2. LT-Cu_3_As Crystal Structure

**Figure 9:**
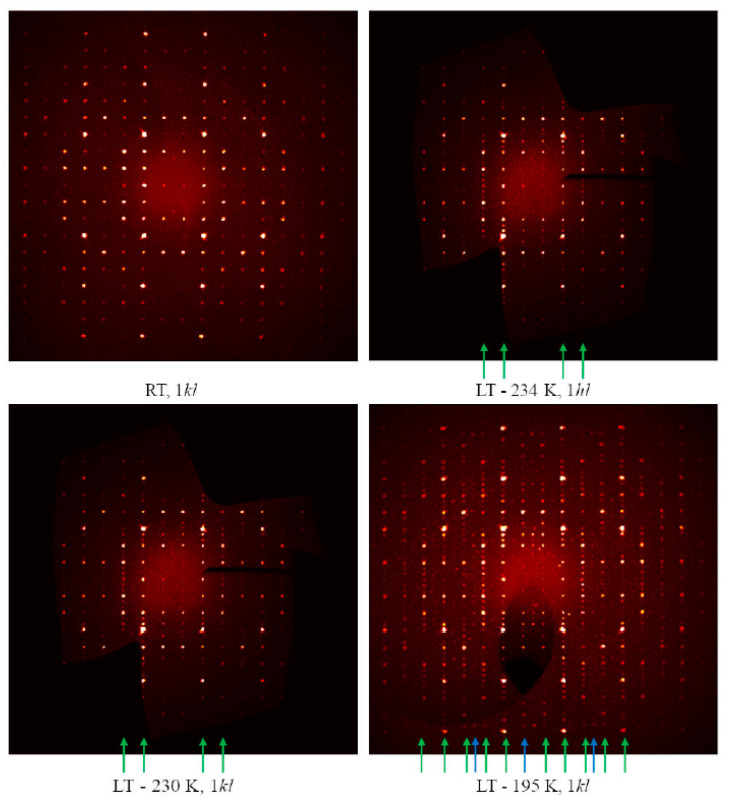
Reconstructed 1*kl* zone intensity profiles for Cu_3_As obtained at different temperatures. The green arrows indicate the lines along which the peaks associated with the formation of the LT polymorph (*hP*72, *a*′ ≈ *a* and *c*′ ≈ 3*c*) appear. Blue arrows indicate a set of very weak super-reflections compatible with an even larger unit cell (*hP*144, *a*′ ≈ 2*a* and *c*′ ≈ 3*c*).

The low-temperature diffraction studies clearly indicate a first phase transition in RT-Cu_3_As occurring at 234 K, which is accompanied by further structural changes. As can be seen from the reconstructed diffraction zones (see Figure 9), the LT modification possesses a unit cell three times larger, with *a*′ ≈ *a* and *c*′ ≈ 3*c*. Moreover, at −78 °C (195 K), further additional and very weak super-reflections become visible. They are likely associated with an additional ordering/structural change of Cu_3_As, which is compatible with a unit cell six times bigger, i.e., *a*′′ ≈ 2*a* and *c*′′ ≈ 3*c*. Hereafter, we will focus on the structural model description of the first transition: from the RT-Cu_3_As form [*hP*24, *a* = 7.1393(1) Å and *c* = 7.3113(1) Å] to the LT-Cu_3_As form [*hP*72, *a* = 7.110(2) and *c* = 21.879(4) Å].

A total of 3490 frames were collected for the −78 °C (195 K) dataset. The integration of the data using a trigonal unit cell yielded a total of 57,491 reflections to a maximum θ angle of 36.39° (0.60 Å resolution), of which 1568 were independent (average redundancy 37, completeness = 99.8%, R_int_ = 7.43%, R_sig_ = 2.10%). The analysis of systematic absences performed using XPREP [15] suggested the only contribution of 6_3_ symmetry element (see Table 5). Consequently, the list of possible space groups became alarmingly long, covering numerous trigonal and hexagonal ones. At this step, it was decided to resolve the crystal structure in the low symmetry space groups. The chemically sound structural model with reasonable interatomic distances (Cu−As interactions ranging from 2.4 to 2.7 Å, and Cu–Cu closest interactions at 2.5 Å) was found in the *P*–3 space group. Generally, 3 As and 14 Cu independent crystallographic sites were assigned, giving 72 atoms total in the unit cell and Cu_3_As as the final stoichiometry. Anisotropic refinement was stable; however, the R1 factor converged at 15% and wR2 at 36%, indicating the necessity of an even deeper structural analysis. A quick check of missed symmetry elements by PLATON [32] efficiently picked up the lacking glide plane, suggesting the *P*–3c1 space group with negligible atoms shift with respect to the *P*–3 starting model. Moreover, the possible twin formation frequently taking place during phase transitions was checked by TwinRotMath routine, which gave a clear indication of the presence of two twin domains. These are related by a 2 fold rotation axis along the *c* direction as a twin law that commonly happens for trigonal/hexagonal cases. The presence of twins explains the difficulties encountered during the analysis of systematic absences related to the presence of -*c*- at previous stage (see Table 5).

Subsequently, the correct space group instruction file was generated. The insertion of TWIN −1 0 0 0 −1 0 0 0 1 and BASF commands [33] led to significant improvement in the least-squares refinement, which dropped to R1 = 6%. The final anisotropic full-matrix least-squares refinement on F^2^ with 58 variables converged at R1 = 4.81%, wR2 = 11.16% and GOF = 1.169. More details on single-crystal refinement are listed in Table 6. In the final model, As atoms are distributed over 2 Wyckoff sites (12*g*, 6*f*), while Cu atoms occupy eight independent positions (2*a*, 4*c*, three 4*d* and three 12*g*). Thus, this structure represents a new structural prototype. Similar to RT-Cu_3_As, in LT-Cu_3_As, only one of the Cu sites (12*g*) is partially occupied (SOF = 0.78, see Table 7) with no other hints on disorder or Cu/As statistical mixture. The refined composition of LT-Cu_3_As perfectly matches that of RT-Cu_3_As, being Cu_2.852(5)_As and Cu_2.881(7)_As, respectively. Similarly to Cu_3−x_As, the analogous off-stoichimetry was recently observed and described in detail for Cu_3−x_P compound [34].

The final standardized atomic coordinates and thermal parameters for the LT-Cu_3_As phase are collected in Table 7 and Table 8, respectively. The corresponding CIF file, available in the Supporting Information, has been deposited at the Cambridge Database with the following depository number: CSD-2235954.

**Figure 10:**
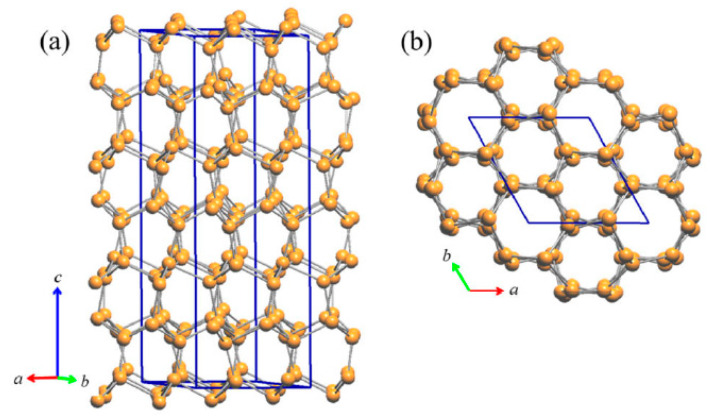
(**a**) Perspective and (**b**) top view of the lonsdaleite type network, which is also preserved in the in the LT structure of Cu_3_As; here, it is formed by Cu6, Cu7 and Cu8 atoms.

**Figure 11:**
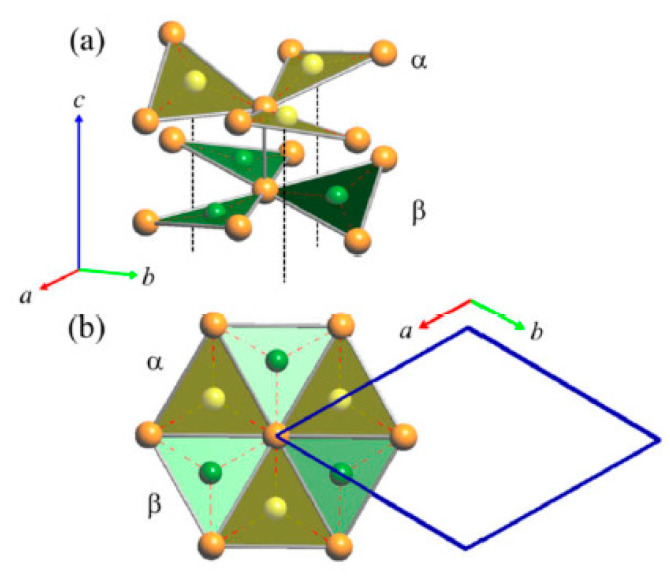
(**a**) Perspective view of the arrangement of the As-centered triangular units in LT—Cu_3_As (only the first two of a total of six different layers are shown); (**b**) top view. Orange and green balls represent Cu and As atoms, respectively.

**Figure 12:**
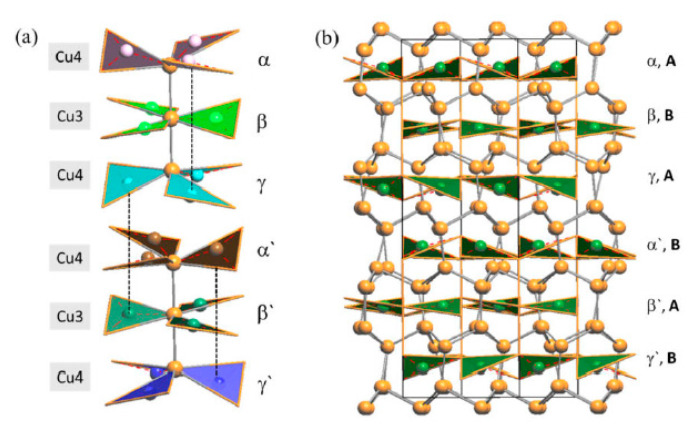
(**a**) A perspective view showing only the assembling of the six different layers of ‘Cu_3_As’-units in the LT—Cu_3_As structure. (**b**) Perspective view of the whole unit cell of the LT structure.

With respect to the RT phase, the structure of LT-Cu_3_As preserves the tridimensional network built up by the Cu sublattice (now formed by the Cu6, Cu7 and Cu8 atoms; all setting in positions 12*g*), but it extends over three of the ‘original’ RT unit cells. A perspective and top view of this structure are depicted in Figure 10. However, in LT-Cu_3−x_As, As atoms occupy two different positions (As1 in 12*g*, As2 in 6*f*), and the number of different layers formed by the As-centered triangular ‘Cu_3_As’ units (always disposed along the *c*-axis) is six (Figure 11). Taking into account the similarity between them and considering also the respective interlayer distances, they can be grouped three by three and labelled accordingly as {α, β, γ} and {α’, β’, γ’} (Figure 12a). A perspective view showing the assembling of the six layers only is depicted in Figure 12b. The main differences between the RT and LT forms of Cu_3_As compound reside in the way these As-containing layers are reciprocally set. In either the first {α, β, γ} or second {α’, β’, γ’} group, the Cu atoms connecting the three triangular ‘Cu_3_As’ units are Cu4, Cu3 and Cu4, respectively. However, the interlayer distances are different and clearly changed: the distances between the two α–β and β–γ layers (same is for the distances between α′–β′ and β′–γ′ layers) are shortened to 2.947 Å, and noticeably increased to 5.045 Å the interlayer γ–α′ distance between the upper and the lower {α, β, γ} and {α’, β’, γ’} groups. Moreover, the structural transition also affects the tilting of the ‘Cu_3_As’-triangular units in each layer as well as their inclination with respect to the *c*-axis: the triangular units face upward in both layers α and α’, they are coplanar and perpendicularly set to the *c*-axis in both layers β and β’, while they face downward in both the layers γ and γ’ (Figure 12a). Further, it must be noted that As atoms are differently set with respect to the plane of the triangular units: they are placed slightly above the plane in layers α and α’, in-plane in the layers β and β’, and slightly below the plane for the layers γ and γ’ (Figure 12b). The value of the lattice parameters (*a* and *c*) and the unit cell volume (V_cell_) as a function of temperature are all collected in Table S4; their trend as a function of temperature is shown in Figures S1 and S2, respectively.

### 3.3. DTA and DSC Calorimetry

**Figure 13:**
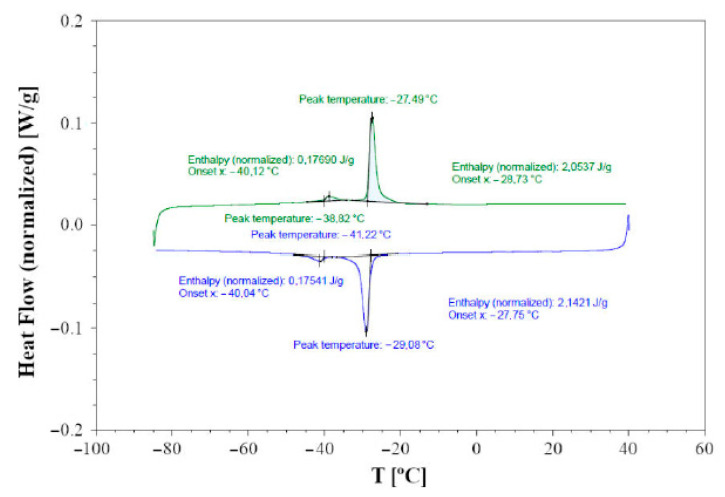
Plot of the DSC data collected on cooling (blue curve) and on heating (green curve) at 5 °C/min on a single crystal of the sample Cu_3_As annealed at 300 °C—13 days. Endothermic heat flow direction is upward.

DTA indicates that Cu_3_As is formed by congruent melting at the temperature of 835 °C, which is slightly higher than the previously reported literature data (827 °C). The DSC calorimetry curves detected on cooling (blue curve) and subsequent heating (green curve) runs at 5 °C/min for a single crystal of the sample 300 °C—20 days are shown in Figure 13. The heat flow direction is upward for endothermic effects and downward for exothermic effects. A rather sharp exothermic peak is observed during cooling, centered around −29 °C, which can be associated with the structural phase transition occurring at the same temperature range, as highlighted by X-ray diffraction. An integration of the main peak associated with the structural phase transition gives an enthalpy difference, ΔH, of approximately 2 J/g. A second clear effect, that despite being weaker is well reversible, appears at about −40 °C, and we believe it is also of a structural nature and related to the doubling of the lattice parameter *a*; however, its origin remains undetermined at present.

### 3.4. Transport and Magnetic Properties

**Figure 14:**
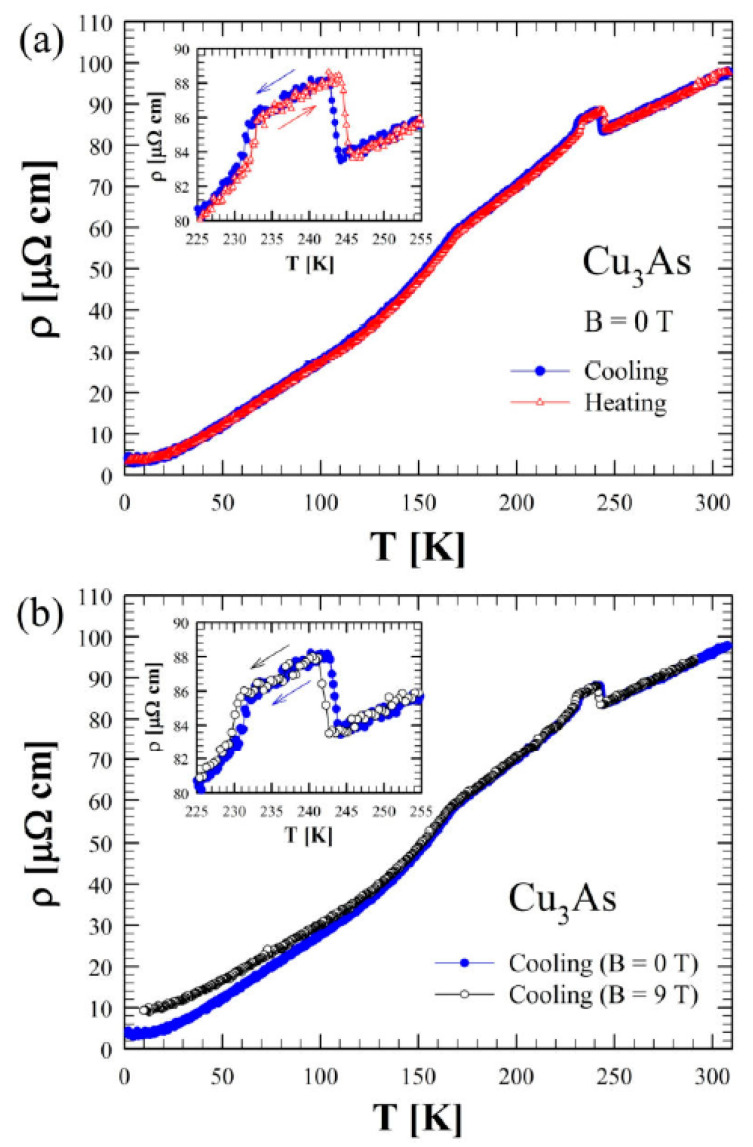
(**a**) Zero-field electrical resistivity as a function of temperature between 2 and 300 K for Cu_3_As measured both on cooling and heating. (**b**) Electrical resistivity measured in zero and under applied magnetic field of 9 *T* (both on cooling) for Cu_3_As. The insets in (**a**,**b**) show a magnification of the data between 225 and 255 K.

**Figure 15:**
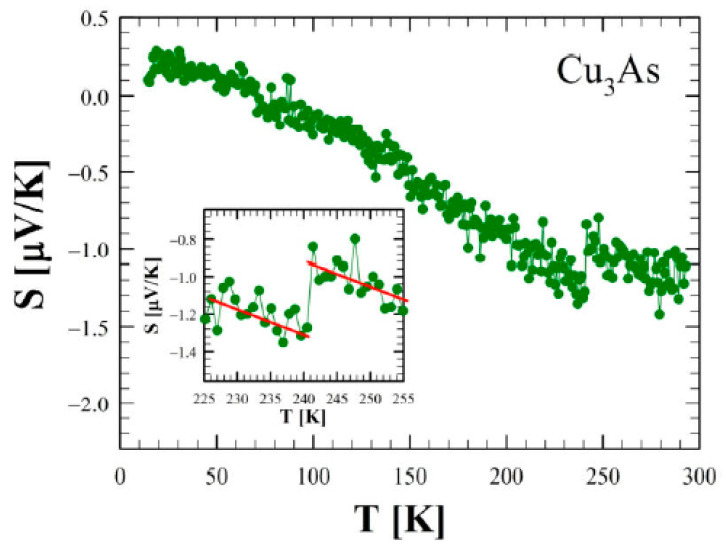
Seebeck coefficient as a function of temperature between 2 and 300 K for Cu_3_As. The inset, which shows a magnification of the data between 225 and 255 K, highlights the discontinuity at about 242 K.

Figure 14a shows the zero-field electrical resistivity as a function of temperature between 2 and 300 K for a single crystal of Cu_3_As (sample 300 °C/20 d). The room temperature value of 100 μΩ cm is of the same order of magnitude as the one reported in the literature for Cu_3−x_P [34]. The bulk resistivity displays an overall metallic behavior with a relative resistivity ratio RRR = 27 and the presence of three evident anomalies. Firstly, at around T = 244 K and T = 231 K, two step-like behaviors in the resistivity appear, which are consistent with the DSC calorimetry peaks. This phenomenology is related to the first-order phase transition, as highlighted by the hysteresis between the data collected upon cooling and upon heating (see the inset of Figure 14a). Interestingly, an applied magnetic field B = 9 T, oriented parallel to the c-axis and perpendicular to the current direction (*a–b* plane), leads to a slight temperature shift of the two transition temperatures with respect to the zero-field curve (inset of Figure 14b). Moreover, there is a net change of slope around T = 167 K. This anomaly is similar to the one reported in the literature for Cu_3−x_P at 180 K [34], but we have not observed any hysteretic behavior in our compound. Although two hitherto Cu_3−x_P polymorphs were probed by temperature-dependent diffraction analysis, and X-ray data have highlighted another first-order phase transition in the sample at a comparable temperature. Figure 15 shows the trend of the Seebeck coefficient (S) as a function of the temperature between 15 K and 290 K for the same Cu_3_As single crystal. At room temperature, we obtained S = −1 μV/K, and the overall negative slope indicates electron-type bulk charge carriers, in contrast with Hall measurements presented in the literature for Cu_3−x_P [34]. On the one hand, the first-order structural transition around T = 243 K is probed by an abrupt jump of 0.3 μV/K (see the magnification of the data in the inset). On the other hand, the two additional anomalies reported in the resistivity cannot be clearly revealed in the Seebeck coefficient. At low temperatures, the thermopower becomes slightly positive, but this is probably linked to the contribution of copper wires used as electric connections. Considering the overall standard behavior of the Seebeck coefficient (*S*) as a function of temperature (*T*), we can use the Mott formula for a parabolic electron band in the semi-classical framework:

(1) $$S=-\frac{\pi^{2}}{3}\frac{k_{B}^{2}T}{eE_{F}}$$

where *E_F_* is the Fermi energy and *e* and *k_B_* are fundamental constants [35]. Using that formula at room temperature, we can estimate that the Fermi level is positioned at ≈7.2 eV in the conduction band.

**Figure 16:**
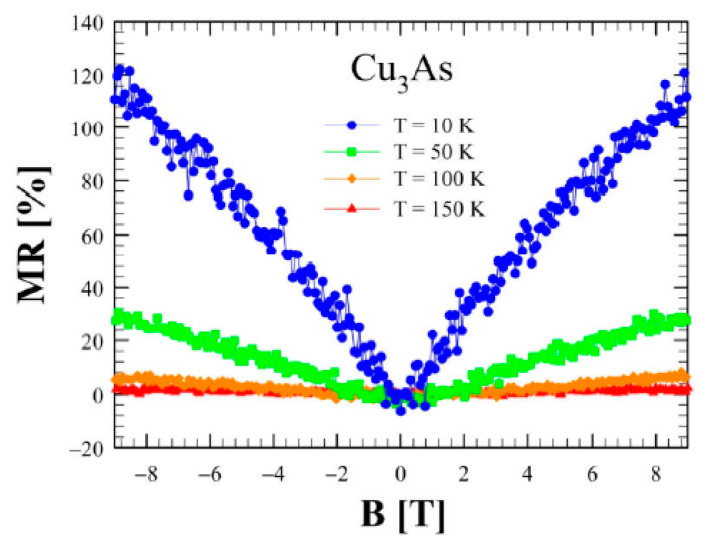
Magnetoresistance as a function of applied magnetic field between 0 and ± 9 *T* at several temperatures (10 K, 50 K, 100 K and 150 K) for Cu_3_As.

Figure 16 shows the magnetoresistance (MR) as a function of the applied magnetic field perpendicular to the *a–b* plane at various temperatures. This quantity presents an intriguing behavior at T = 10 K: at low fields MR is linear as a function of the magnetic field, whereas at higher fields it seems to approach a saturation, reaching a large value of 120% at 9 T. Remarkably, this value is one order of magnitude larger than the one reported for Cu_3−x_P [36], and in general, it is much higher than in standard metal. This behavior is replicated at T = 50 K and at T = 100 K, but the signal is attenuated while increasing the temperature. Above T = 167 K, where the slope change in the resistivity is observed, MR becomes negligibly small. The phenomenology described at low temperatures is neither linked to the parabolic behavior of a standard metal nor to that of a multi-band material, and further ab-initio calculations of the band structure could elucidate more in detail the nature of this anomalous response. In particular, the overall metallic nature of Cu_3_As, both at RT and LT, is presumably related and due to the 3D Cu lonsdaleitic sublattice. Such as Cu-network, presumably presenting bonds with a more covalent-metallic character, likely remains more rigid and less sensitive to the lowering of temperature; unlike to the 2D As-containing layers that should be characterized by more covalent-ionic bounds.

**Figure 17:**
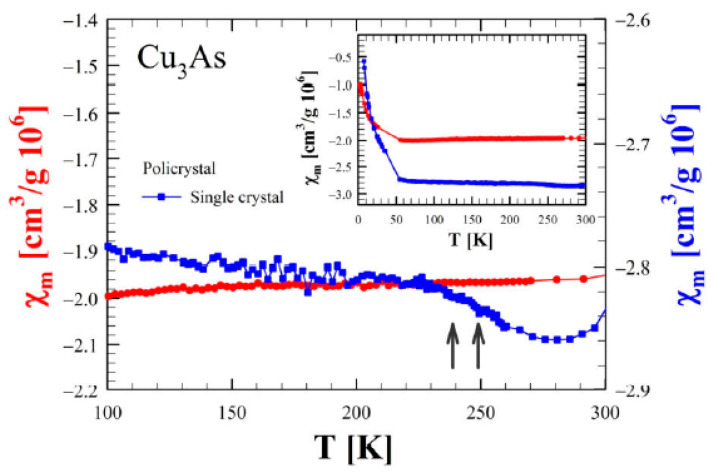
Magnetic susceptibility (in CGS units) as a function of temperature between 100 and 300 K for both single crystal (blue line and scale on the right) and a polycrystalline sample (red line and scale on the left) of Cu_3_As. Measurements were performed using an external magnetic field of 1 *T* oriented perpendicular to the *a–b* plane for the single crystal. The inset shows an overview of the data from 5 to 300 K.

The temperature dependence of the mass magnetic susceptibility is shown in Figure 17 for both a single crystal and a polycrystalline sample of Cu_3_As. In both cases, the magnetic susceptibility displays an overall constant diamagnetic response with a small superimposed paramagnetic component visible at low temperatures. Considering the nature of the single crystal, the magnetic response is smaller than the one observed in the polycrystal, but there is a slight increase in the signal for temperatures greater than T = 230 K that cannot be revealed in the polycrystal, and that is likely related to the order transitions reported before.

## 4. Conclusions

In this study, we reinvestigated the crystal structure of the binary Cu_3_As phase by single-crystal and powder X-ray diffraction. Rather than being in the anti HoH_3_-type (or anti LaF_3_-type) (*hP*24, *P*3*c*1, No. 165) as indicated in literature, this compound crystallizes in the acentric hexagonal Cu_3_P prototype (*hP*24, *P*6_3_*cm*, No. 185), with RT lattice parameters: *a* = 7.1393(1) Å and *c* = 7.3113(1) Å. A slight understoichiometry is found in one out of the four Cu atomic sites, which leads to the final refined composition Cu_2.882(1)_As. Our powder X-ray diffraction confirmed both the structural prototype as well as the fractional occupation of Cu from samples obtained from synthesis at lower temperatures (300 °C and 350 °C). We disprove a change in the crystal structure (from γ’ to γ) above room temperature, which was actually reported in the phase diagram. Instead, single-crystal X-ray diffraction below room temperature highlights a first-order structural transition to a trigonal LT superstructure polymorph of Cu_3_As (*hP*72, with space-group *P*–3*c*1), at T ≈ −30 °C (243 K), with *a*′ ≈ *a* and *c*′ ≈ 3*c* [*a* = 7.110(2) Å and *c* = 21.879(4) Å]. This transition is then likely followed by an additional structure change/reordering to a unit cell where *a*′′ ≈ 2*a* and *c*′′ ≈ 3*c*, which is observed when lowering the temperature up to T = −118 °C (155 K). However, further synchrotron diffraction works down to lower temperatures are necessary to study and refine this latter structure. The first low-temperature structural change is clearly detected by a distinct thermal effect in differential scanning calorimetry, both in single crystals and bulk material, with an associated enthalpy difference, ΔH_(TR)_, of approximately 2 J/g. The nature of a second and weaker, reversible, thermal effect at about −40 °C (233 K) remains uncertain; we suggest it likely has a structural origin and might be related to the doubling of the lattice parameter *a* of the unit cell, which, however, becomes detectable by our XRD apparatus only at lower temperatures. A typical metallic behavior for Cu_3_As is outlined by low-temperature electrical resistivity measurements, and the data nicely show clear anomalies in correspondence with the structural changes. From the analysis of the Seebeck coefficient as a function of temperature, we have determined a conduction of the *n*-type. These measurements again probe the first structural transition. The temperature dependence of the magnetic susceptibility, measured for both a single crystal and a polycrystalline sample of Cu_3_As, displaying an overall constant diamagnetic response, demonstrated that this compound is a diamagnetic material.

## Figures and Tables

**Table 1:** Crystal data and structure refinement details for the RT-Cu_3−x_As compound.

Compound	Cu_2.881(7)_As
Temperature [K]	293(2) K
Formula weight [g/mol]	265.54
Structural prototype	Cu_3_P
Pearson symbol	*hP*24-0.71
Crystal system	Hexagonal
Space group	*P*6_3_*cm* (No. 185)
*a* [Å]	7.1407(3)
*c* [Å]	7.3057(6)
Unit cell volume [Å^3^]	322.61(4)
Unit formula per cell, *Z*	6
Calculated density, ρ [g/cm^3^]	7.97
Absorption coefficient, µ [mm^−1^]	43.06
*F*(000)	700
Crystal description	Irregular form, metallic luster
Theta range [°]	3.3° ≤ ϑ ≤ 45.2°
Index ranges *h*, *k*, *l*	−14 ≤ *h* ≤ 14
	–14 ≤ *k* ≤ 14
	–14 ≤ *l* ≤ 14
Reflections collected	18668
Absorption correction	Multiscan
Refinement method	Full-matrix least-squares on *F*^2^
Data/parameter	991/26
Absolute structure parameter	0.11(12)
Goodness of fit on *F*^2^	1.08
Final *R* indices [*I* > 2σ(*I*)]	*R*1 = 0.0349, *wR*2 = 0.0927
*R* indices (all data)	*R*1 = 0.0460, *wR*2 = 0.0979
*R*_int_/*R*_sym_	0.0748/0.0255
Largest diff. peak and hole [e^–^/Å^3^]	+1.82, –2.27

**Table 2:** Standardized atomic coordinates and equivalent displacement parameters (*U*_eq_) for RT-Cu_2.881(7)_As.

Atom	Site	Atomic Coordinates			Occ.	*U*_eq_ [Å^2^]
		*x/a*	*y/b*	*z/c*		
As	6*c*	0.33416(10)	0	0.0823(4)	1	0.01172(13)
Cu1	2*a*	0	0	0.00000 *	1	0.0212(3)
Cu2	6*c*	0.7121(2)	0	0.2504(4)	1	0.0239(2)
Cu3	4*b*	1/3	2/3	0.1550(5)	1	0.0373(5)
Cu4	6*c*	0.36811(19)	0	0.4129(4)	0.881(7)	0.0271(4)

(*) chosen as the fixed position because of the floating origin along [001] for *P*6_3_*cm* space group.

**Table 3:** Anisotropic displacement parameters for RT-Cu_2.881(7)_As as obtained from single crystal investigation.

Atom	Site	*U*_11_	*U*_22_	*U*_33_	*U*_23_	*U*_13_	*U*_12_
As	6*c*	0.01100(17)	0.0118(2)	0.0124(2)	0	0.00053(14)	0.00590(11)
Cu1	2*a*	0.0129(3)	0.0129(3)	0.0367(8)	0	0	0.00646(15)
Cu3	6*c*	0.0331(4)	0.0156(4)	0.0164(4)	0	0.0034(5)	0.00779(19)
Cu2	4*b*	0.0149(3)	0.0149(3)	0.0828(16)	0	0	0.00744(16)
Cu4	6*c*	0.0232(4)	0.0535(10)	0.0142(5)	0	−0.0032(4)	0.0268(5)

**Table 4:** Interatomic distances corresponding to the first coordination sphere for d_obs_/Σ*r*_M_ ≤ 1.16 in Cu_2.882(1)_As, as obtained from single crystal analysis.

Central Atom	Ligands	d [Å]	d_obs_/Σ*r*_M_	Polyhedron
**As** CN = 11	1 Cu1	2.4605(11)	0.922	Pseudo Frank–Kasper polyhedra As@Cu_11_
	2 Cu2	2.4361(8)	0.913	
	1 Cu3	2.4469(18)	0.918	
	2 Cu3	2.5541(12)	0.957	
	1 Cu3	2.9652(16)	1.111	
	1 Cu4	2.4277(18)	0.910	
	1 Cu4	2.4601(16)	0.922	
	2 Cu4	2.8037(10)	1.051	
**Cu1** CN = 12	3 As	2.4605(10)	0.922	Icosahedron Cu@Cu_9_As_3_
	3 Cu3	2.7483(22)	1.075	
	3 Cu3	2.7516(21)	1.076	
	3 Cu4	2.7044(12)	1.058	
**Cu2** CN = 12	3 As	2.4361(9)	0.913	Icosahedron Cu@Cu_9_As_3_
	3 Cu3	2.6511(12)	1.037	
	3 Cu4	2.8751(21)	1.125	
	3 Cu4	2.9470(21)	1.153	
**Cu3** CN = 12	1 As	2.4469(18)	0.918	Icosahedron Cu@Cu_8_As_4_
	2 As	2.5541(10)	0.957	
	1 As	2.9652(16)	1.111	
	1 Cu1	2.7483(23)	1.075	
	1 Cu1	2.7516(23)	1.076	
	2 Cu2	2.6511(12)	1.037	
	2 Cu4	2.6722(8)	1.046	
	1 Cu4	2.5313(15)	0.990	
	1 Cu4	2.7284(19)	1.068	
**Cu4** CN = 13	1 As	2.4277(18)	0.910	Pseudo Frank–Kasper polyhedra Cu@Cu_9_As_4_
	1 As	2.4601(16)	0.922	
	2 As	2.8037(11)	1.051	
	1 Cu1	2.7044(15)	1.058	
	2 Cu2	2.8751(20)	1.125	
	2 Cu2	2.9740(21)	1.153	
	1 Cu3	2.5313(15)	0.990	
	2 Cu3	2.6722(13)	1.046	
	1 Cu3	2.7284(19)	1.068	

**Table 5:** Systematic absence exceptions for LT-Cu_3−x_As dataset.

	6^1^/6^5^	6^2^/3^1^	6^3^	-*c*-	--*c*
**Total**	218	173	126	5313	2881
***N* (*I* > 3)**	101	89	14	631	1914
**〈*I*** **〉**	6.0	7.5	0.2	0.4	16.6
**〈*I/σ*** **〉**	5.5	6.5	1.1	1.3	6.6

**Table 6:** Crystal data and structure refinement details for the LT-Cu_3−x_As compound.

Compound	Cu_2.852(5)_As
Temperature [K]	195(2)
Formula weight [g/mol]	256.12
Structural prototype	Own
Pearson symbol	*hP*72
Crystal system	Trigonal
Space group	*P*–3c1 (No. 165)
*a* [Å]	7.110(2)
*c* [Å]	21.879(4)
Unit cell volume [Å^3^]	957.8(6)
Unit formula per cell, *Z*	18
Calculated density, ρ [g/cm^3^]	7.99
Absorption coefficient, µ [mm^−1^]	43.21
*F*(000)	2083
Crystal description	Irregular form, metallic luster
Theta range [°]	2.8° ≤ ϑ ≤ 36.4°
Index ranges *h*, *k*, *l*	−11 ≤ *h* ≤ 11
	–11 ≤ *k* ≤ 11
	–36 ≤ *l* ≤ 36
Reflections collected	57491
Absorption correction	Multiscan
Refinement method	Full-matrix least-squares on *F*^2^
Data/parameter	1568/58
Twin law	−1 0 0 0 −1 0 0 0 −1
BASF	0.60
Goodness of fit on *F*^2^	1.17
Final *R* indices [*I* > 2σ(*I*)]	*R*1 = 0.0481, *wR*2 = 0.1103
*R* indices (all data)	*R*1 = 0.0510, *wR*2 = 0.1116
*R*_int_/*R*_sym_	0.0743/0.0210
Largest diff. peak and hole [e^–^/Å^3^]	+4.03, –4.11

**Table 7:** Standardized atomic coordinates and equivalent displacement parameters (Ueq) for LT-Cu_2.825(5)_As.

Atom	Site	Atomic Coordinates			Occ.	*U*_eq_ [Å^2^]
		*x/a*	*y/b*	*z/c*		
As1	12*g*	0.32922(14)	0.32932(15)	0.07954(3)	1	0.01017(13)
As2	6*f*	0.34120(17)	0	1/4	1	0.00890(16)
Cu1	4*d*	1/3	2/3	0.05029(8)	1	0.0138(3)
Cu2	4*d*	1/3	2/3	0.39253(9)	1	0.0147(3)
Cu3	2*a*	0	0	1/4	1	0.0196(5)
Cu4	4*c*	0	0	0.11530(10)	1	0.0171(3)
Cu5	4*d*	1/3	2/3	0.22502(12)	1	0.0232(4)
Cu6	12*g*	0.06180(2)	0.39440(2)	0.13869(5)	1	0.0218(2)
Cu7	12*g*	0.34900(2)	0.30257(16)	0.19163(5)	1	0.01458(19)
Cu8	12*g*	0.34710(2)	0.03300(2)	0.02891(5)	0.778(5)	0.0135(3)

**Table 8:** Anisotropic displacement parameters for the LT-Cu_2.825(5)_As, as obtained from single crystal investigation.

Atom	Site	*U*_11_	*U*_22_	*U*_33_	*U*_23_	*U*_13_	*U*_12_
As1	12*g*	0.0087(3)	0.0092(3)	0.0126(3)	−0.0027(2)	0.0003(2)	0.0045(3)
As2	6*f*	0.0110(4)	0.0083(3)	0.0083(3)	−0.00024(18)	−0.0005(4)	0.0055(2)
Cu1	4*d*	0.0112(4)	0.0112(4)	0.0189(7)	0.000	0.000	0.0056(2)
Cu2	4*d*	0.0114(4)	0.0114(4)	0.0213(7)	0.000	0.000	0.0057(2)
Cu3	2*a*	0.0071(5)	0.0071(5)	0.0446(15)	0.000	0.000	0.0036(3)
Cu4	4*c*	0.0122(4)	0.0122(4)	0.0269(8)	0.000	0.000	0.0061(2)
Cu5	4*d*	0.0102(4)	0.0102(4)	0.0494(12)	0.000	0.000	0.0051(2)
Cu6	12*g*	0.0358(6)	0.0331(6)	0.0145(4)	0.0085(4)	0.0106(4)	0.0307(5)
Cu7	12*g*	0.0175(4)	0.0100(4)	0.0150(3)	0.0044(3)	0.0003(4)	0.0059(4)
Cu8	12*g*	0.0116(6)	0.0247(7)	0.0082(4)	0.0035(5)	0.0027(4)	0.0121(6)

## Supplementary Materials

The following supporting information can be downloaded at: https://www.mdpi.com/article/10.3390/ma16062501/s1.
