# Supplementary material for: Cu3As: Uncommon Crystallographic Features, Low-Temperature Phase Transitions, Thermodynamic and Physical Properties
Source: Materials (Basel). 2023 Mar 21;16(6):2501. doi: 10.3390/ma16062501 (PMC10051385; doi:10.3390/ma16062501)
Supplement: Supplementary file 1 [file materials-16-02501-s001.zip › materials-2256859-supplementary.pdf]

Cu_3As: Uncommon Crystallographic Features, Low-Temperature Phase Transitions, Thermodynamic and Physical Properties

M. Mödlinger [1], A. Provino [1], P. Solokha [1], F. Caglieris [2,3], M. Ceccardi [2], D. Macciò [1],
M. Pani [1], C. Bernini [3], D. Cavallo [1], A. Ciccioli [4], P. Manfrinetti [1,3,]*

[1] *Department of Chemistry, University of Genoa, 16146 Genoa, Italy*
[2] *Department of Physics, University of Genoa, 16146 Genoa, Italy*
[3] *Institute SPIN-CNR, 16152 Genoa, Italy*
[4] *Department of Chemistry, Sapienza University of Rome, 00185 Rome, Italy*

Supplementary Materials

Table S1. Overview of the binary alloys prepared in this work, their thermal treatment and analyses carried out.

Nominal composition	Thermal treatment	Quenching	X-ray diffraction	SEM-EDS	Metallography	Phase analysis
**Cu_3As**	300°C - 20 days	air	single crystal	✓	-	$Cu_3As + Cu_5As_2$
	350°C - 13 days	air	single crystal, powder	✓	✓	Cu_3As
	400°C - 4.5 days	air	powder	-	-	Cu_3As
	400°C – 14 days	no	powder	✓	✓	Cu_3As
	500°C - 16 days	air	powder	✓	✓	$Cu_3As + Cu_5As_2$
	500°C – 16 days → 750°C - 1 day	water	powder	-	-	$Cu_3As + Cu_5As_2$
	650°C - 19 days	water	powder	-	-	Cu_3As
	650°C - 19 days → 750°C – 1 day	air	powder	-	-	$Cu_3As + Cu_5As_2$
	750°C - 15 days	air	powder	✓	✓	Cu_3As
	750°C – 15 days → 300°C - 12 days	air	powder	-	✓	Cu_3As

Table S2. Refined atomic coordinates (standardized setting) for Cu_3As (Cu_3P-type, *hP*24, *P*6_3*cm*, No. 185) as obtained from the Rietveld refinement fit on the X-ray powder pattern of the sample annealed at 350°C for 13 days (Cu3As_8_350C). The sample is single phase containing the **$Cu_{2.885(1)}As$** compound.

Atom	Wyckoff site	*x*	*y*	*z*	Occ.	B_{iso} [Å^2]	U_{iso} [Å^2]
As	6*c*	0.3348(2)	0	0.082(3)	1	0.62(1)	0.0078(1)
Cu1	2*a*	0	0	0.012(3)	1	1.73(4)	0.0219(1)
Cu2	4*b*	1/3	2/3	0.164(3)	1	0.86(2)	0.0109(1)
Cu3	6*c*	0.7125(1)	0	0.250(3)	1	1.93(3)	0.0244(1)
Cu4	6*c*	0.3682(1)	0	0.4109(1)	0.885(1)	2.23(3)	0.0282(1)

Final refined stoichiometry: **$Cu_{2.885(1)}As$** (Cu_3P-type, *hP*24, *P*6_3*cm*)

a = 7.1393(1) Å, c = 7.3113(1) Å, V_{cell} = 322.73(1) Å^3

R_B = 1.75 %, R_F = 1.98 %, R_{wp} = 6.87 %, χ^2 = 8.44

Table S3. Refined atomic coordinates (standardized setting) for Cu_3As (Cu_3P-type, *hP*24, *P*6_3*cm*, No. 185) as obtained from the Rietveld refinement fit on the X-ray powder pattern of the sample Cu_3As annealed at 400°C – 14 days. The sample is single phase containing the **Cu_3As** compound.

Atom	Wyckoff site	*x*	*y*	*z*	Occ.	B_{iso} [Å^2]	B_{iso} [Å^2]
As	6*c*	0.3378(1)	0	0.0672(1)	1	0.601(2)	0.0076(1)
Cu1	2*a*	0	0	0.0000(*)	1	3.347(9)	0.0424(1)
Cu2	4*b*	1/3	2/3	0.1489(1)	1	3.843(6)	0.0487(1)
Cu3	6*c*	0.7106(1)	0	0.2343(1)	1	1.492(5)	0.0189(1)
Cu4	6*c*	0.3624(1)	0	0.3949(1)	1	3.070(6)	0.0389(1)

Final refined stoichiometry: **Cu_3As** (Cu_3P-type, *hP*24, *P*6_3*cm*)

a = 7.1357(1) Å, c = 7.3077(1) Å, V_{cell} = 322.240(2) Å^3

R_B = 0.38 %, R_F = 0.50 %, R_{wp} = 3.56 %, χ^2 = 2.79

(*) chosen as the fixed position (not refined)

Table S4. Single-crystal low-temperature data collected on a crystal picked up from the sample annealed at 350°C for 13 days.

	T [K] measured	T [K] corrected	*a* [Å]	*c* [Å]	V_{cell} [Å^3]	No. peaks (*)
Data taken on cooling	123.0	154.7	7.1123(13)	21.917(3)	960.1(3)	1657
	170.0	191.9	7.100(2)	21.870(5)	954.9(5)	836
	178.0	198.3	7.1048(14)	21.880(4)	956.5(3)	980
	183.0	202.2	7.1086(10)	21.902(2)	958.5(2)	1200
	188.0	202.2	7.1099(11)	21.910(2)	959.2(2)	1400
	193.0	210.1	7.1095(12)	21.903(3)	958.8(3)	1400
	198.0	214.1	7.1124(11)	21.909(3)	959.8(3)	1400
	203.0	218.0	7.1158(11)	21.925(3)	961.4(2)	1400
	208.0	222.0	7.1173(11)	21.928(2)	962.0(2)	1410
	213.0	225.9	7.1140(12)	21.916(3)	960.6(3)	1400
	218.0	229.9	7.1222(12)	21.943(3)	964.0(3)	1370
	223.0	233.9	7.1100(13)	21.886(3)	958.1(3)	1000
Data taken on heating	228.0	237.8	7.1189(13)	7.3039(9)	320.56(9)	1100
	238.0	245.7	7.1205(16)	7.3039(12)	320.71(12)	1100
	300.0	294.8	7.1407(3)	7.3057(6)	322.61(4)	900
	323.0	313.0	7.1460(20)	7.3240(14)	323.89(14)	1050
	348.0	332.8	7.106(3)	7.2806(17)	318.36(17)	1050
	373.0	352.5	7.124(3)	7.2970(18)	320.68(19)	1030

(*) Number of peaks considered for unit-cell determination

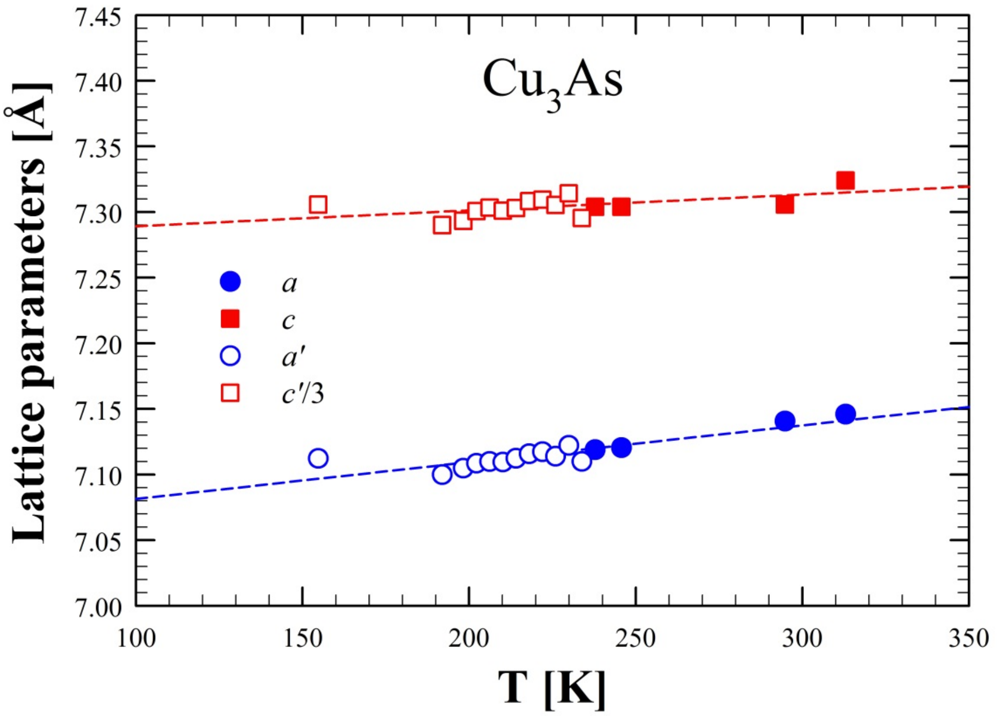

Figure S1. Trend of the lattice parameters as a function of temperature in the range 100–400 K for the high- and low-temperature forms of the Cu_3As intermetallic.

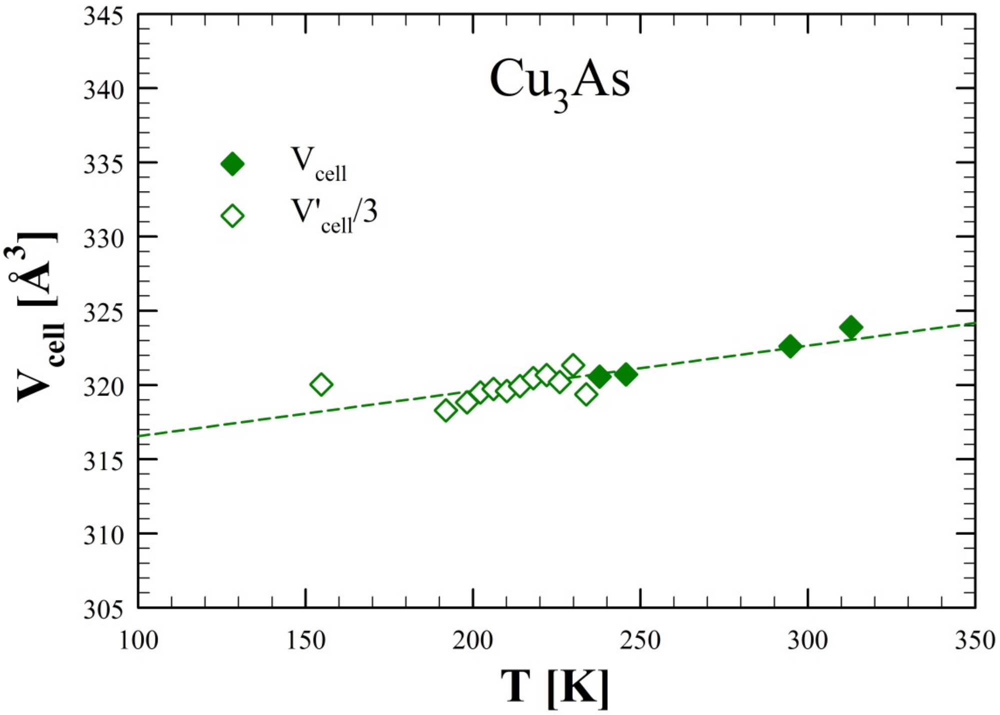

Figure S2. Trend of the unit cell volume as a function of temperature in the range 100–400 K for the high- and low-temperature forms of the Cu_3As intermetallic.
